# From Salt to Stroke—Evaluation of a Media Campaign for Sodium Reduction in Philadelphia

**DOI:** 10.3389/fpubh.2020.619261

**Published:** 2021-01-15

**Authors:** Ann C. Klassen, Suruchi Sood, Amber Summers, Udara Perera, Michelle Shuster, Jessica P. Lopez, Andrea McCord, Jared Stokes, Joann White, Amanda Wagner

**Affiliations:** ^1^Department of Community Health and Prevention, Drexel University Dornsife School of Public Health, Philadelphia, PA, United States; ^2^Center for Communication Programs, Johns Hopkins Bloomberg School of Public Health, Baltimore, MD, United States; ^3^Philadelphia Department of Public Health, Philadelphia, PA, United States

**Keywords:** sodium reduction, health communication, evaluation, health disparities, cardiovascular disease, mass media

## Abstract

Excess dietary sodium contributes to the burden of chronic disease, including cardiovascular disease and stroke. Media-based health education campaigns are one strategy to raise awareness among populations at greater risk for stroke, including African Americans. During 2014–2015, the Philadelphia Department of Public Health conducted a health education campaign using radio, print news, and transit ads, to promote awareness of the link between dietary sodium, hypertension and stroke, and encourage reduced consumption of high sodium foods. Using a repeated cross-sectional design, street intercept surveys were conducted with ~400 Philadelphia residents representing the campaign's priority audience (African Americans ages 35–55) before and 6–13 weeks after the campaign, to evaluate both process (campaign exposure) and impact (recall of key health messages). Thirty percent of post-campaign respondents reported familiarity with one of the most engaging radio spots, and 17% provided accurate unaided recall of its key content, with greater recall among older respondents and frequent radio listeners. Forty-one percent of post-campaign respondents named stroke as a consequence of excess salt consumption, compared to only 17% of pre-campaign respondents, with greater awareness of the salt-stroke connection among those accurately recalling the radio spot from the campaign. Results suggest that priority populations for sodium reduction can be effectively reached through radio and transit campaigns. From a pragmatic perspective, street intercept surveys may offer one low resource strategy for evaluating public health education campaigns conducted by local health departments, especially among urban populations.

## Introduction

Globally, strategies to address the burden of non-communicable diseases have a growing focus on dietary behavior change, including recommendations from the World Health Organization (1) to limit sodium consumption to 2 grams per day. Most Americans consume far more than the recommended level of salt (2), and therefore almost everyone would benefit from reducing dietary sodium to decrease risk for chronic diseases, including hypertension and stroke (3, 4). However, priority populations for sodium reduction include persons over age 50, African Americans and those with histories of cardiovascular disease (5).

Stroke is the fifth leading cause of death in the US (6), as well as a cause of significant disability, with severe disparities by race and ethnicity. Racial minorities, including African Americans, have higher rates of both fatal and non-fatal stroke, experience stroke at earlier ages, and bear more significant post-stroke disability (5). Despite the significant burden of stroke, however, studies show that more adults are aware of excess sodium's connection to hypertension, than its connection to stroke (7).

Multi-level approaches to sodium reduction focus on changing both supply (i.e., food production) and demand for salt in foods (8, 9). One review of 70 studies globally found that “upstream” approaches (i.e., manufacturing policies and taxation) as well as multi-component strategies (food labeling and media campaigns) demonstrated significant effects. However, “downstream” individually-focused approaches had less population-level impact (10).

Targeted health communication campaigns have proven to be successful at increasing awareness of health risks associated with dietary behaviors, and motivating behavior change in key audiences (11, 12). Health communication campaigns can be cost effective ways to reach broad audiences with actionable messages (13), especially as part of multi-level strategies, including changes in the food environment (i.e., promoting lower sodium options in key venues). Because most salt is added during processing and preparation, rather than when eating, one important strategy is to increase consumer awareness of “hidden” salt in processed and prepared foods (14).

## Context and Purpose

To address excess sodium and related stroke risk in Philadelphia, a city with substantial chronic disease burden and health disparities, the Philadelphia Department of Health created and carried out a salt reduction media campaign during 2014–2015. The campaign was designed to leverage other salt reduction activities in Philadelphia, including a Healthy Chinese Take-Out Initiative (15) as well as broader nutrition and physical activity programs (www.phila.gov/programs/get-healthy-philly/), and was funded in part through the Centers for Disease Control and Prevention (CDC)'s Community Transformation Grant initiative.

Formative research activities to prepare for the media campaign included a review of the literature on media campaigns for sodium reduction, as well as primary data collection, through focus groups with the target audiences, to identify knowledge, attitudes and behaviors related to sodium, hypertension, and stroke. Formative findings indicated that respondents were familiar with stroke (most often debilitating characteristics of impaired speech and movement) but had less understanding of the possible risk through uncontrolled hypertension and cardiovascular disease. As a result, although participants knew the connection between salt and hypertension, few connected excess dietary sodium to stroke risk.

These findings informed the development of a media campaign by a professional media partner. In June 2014, the Department of Public Health launched a media campaign, delivering salt reduction/stroke prevention messages using radio, transit billboards, and local weekly print news. An initial campaign ran from June through October, 2014, and a second campaign ran from January through April, 2015.

### Campaign Content

**Figure 1:**
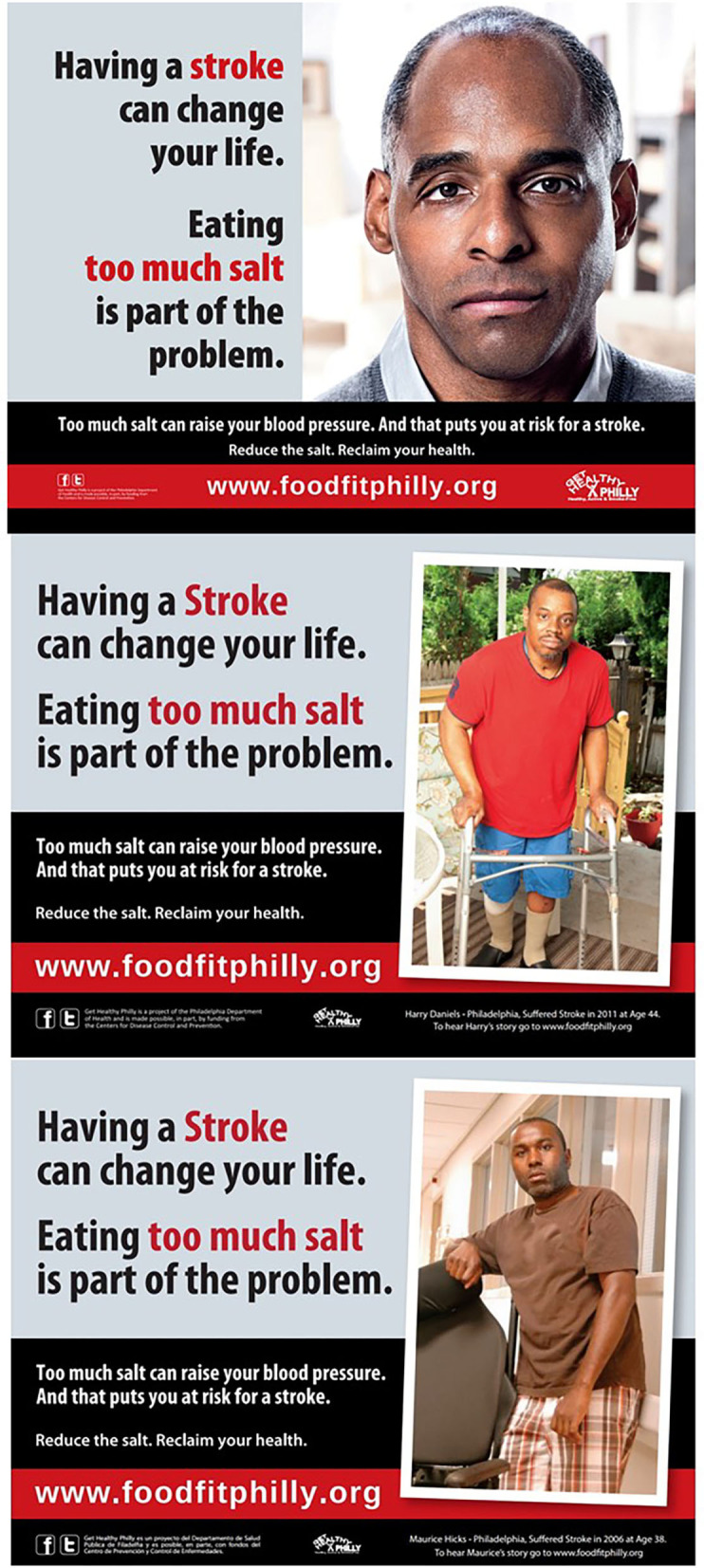
Print advertisements used in salt media campaign.

Three print ads (Figure 1) featured African-American men who had experienced stroke, and described resulting disability. One image featured an actor, while the other two featured Philadelphia residents who had experienced stroke. (Written informed consent is not required to display these images as Figure 1, because these individuals knowingly participated in creating the campaign, and verbally consented to their images appearing in the publicly displayed campaign ads and related promotional activities).

In addition to print ads, two radio advertisements appeared throughout the year on six local radio stations popular with the target audience (e.g., gospel, jazz, rhythm and blues, and an African-American focused talk radio station). “Stroke” featured men and women speaking about the experience of their strokes, and how they are now reducing sodium to avoid future health issues. “Mom Says” featured a young girl playing basketball with her father. He offers her “cheesesteaks and fries” if she wins, but she responds with cautions about high sodium meals, his hypertension and its link to stroke (audio and print images available from the authors on request).

### Evaluation Design

Prior to campaign launch, we designed and conducted central location intercept surveys (16) with Philadelphia residents (*N* = 400), who self-identified at screening as African-American Philadelphia residents age 35–55. Respondents were recruited at 16 outdoor locations throughout the City, including transit hubs, shopping venues and parks, with locations purposively selected to represent both center city commercial settings as well as geographically dispersed predominantly African-American residential neighborhoods. Respondents received a $10 Walmart gift card on the spot for survey completion. Teams with two male and female interviewers approached potentially eligible respondents and asked a brief set of questions to screen for Philadelphia residency, age and ethnicity. In addition, to maximize the likelihood of campaign exposure, respondents were asked how often they used public transportation, listened to radio, and read the weekly newspapers where the campaign appeared. Verbal informed consent was obtained from eligible and willing participants.

The 10-min, interviewer-administered anonymous survey captured knowledge, attitudes and practices related to diet, sodium consumption, and chronic diseases, including hypertension and stroke, as well as socio-demographic (age, zip code of residence, household composition, education, and work status) and health information [self-rated health, history of hypertension, and interviewer-estimated body size using a well-validated visual rating scale of 10 body sizes (17)]. Baseline survey items were designed to measure key message content of the pending campaign, to establish pre-campaign benchmarks. Sample questions that were asked of respondents include: Do you think most salt in our food comes from food we eat at home, or food that we eat outside our homes?; what are some of the health problems you know of that can come from having too much salt in your diet?; can you name some foods that have a lot of salt in them?; and what are some of the problems people can have after a stroke?

Follow-up surveys collected at 6–13 weeks after completion of the second campaign with similarly recruited intercept participants used many of the same measures of salt and stroke knowledge, attitudes and behaviors, and also measured both aided and unaided recall of key campaign elements. For example, after asking respondents to describe any health-related transit posters or newspaper ads they had recently seen (unaided recall), they were next shown large visuals of each specific campaign poster and asked if they had ever seen them, and where (aided recall). The two surveys provided cross-sectional comparison data, and differences between pre- and post-campaign responses on campaign-specific health messages served as an indicator of campaign impact.

**Figure 2:**
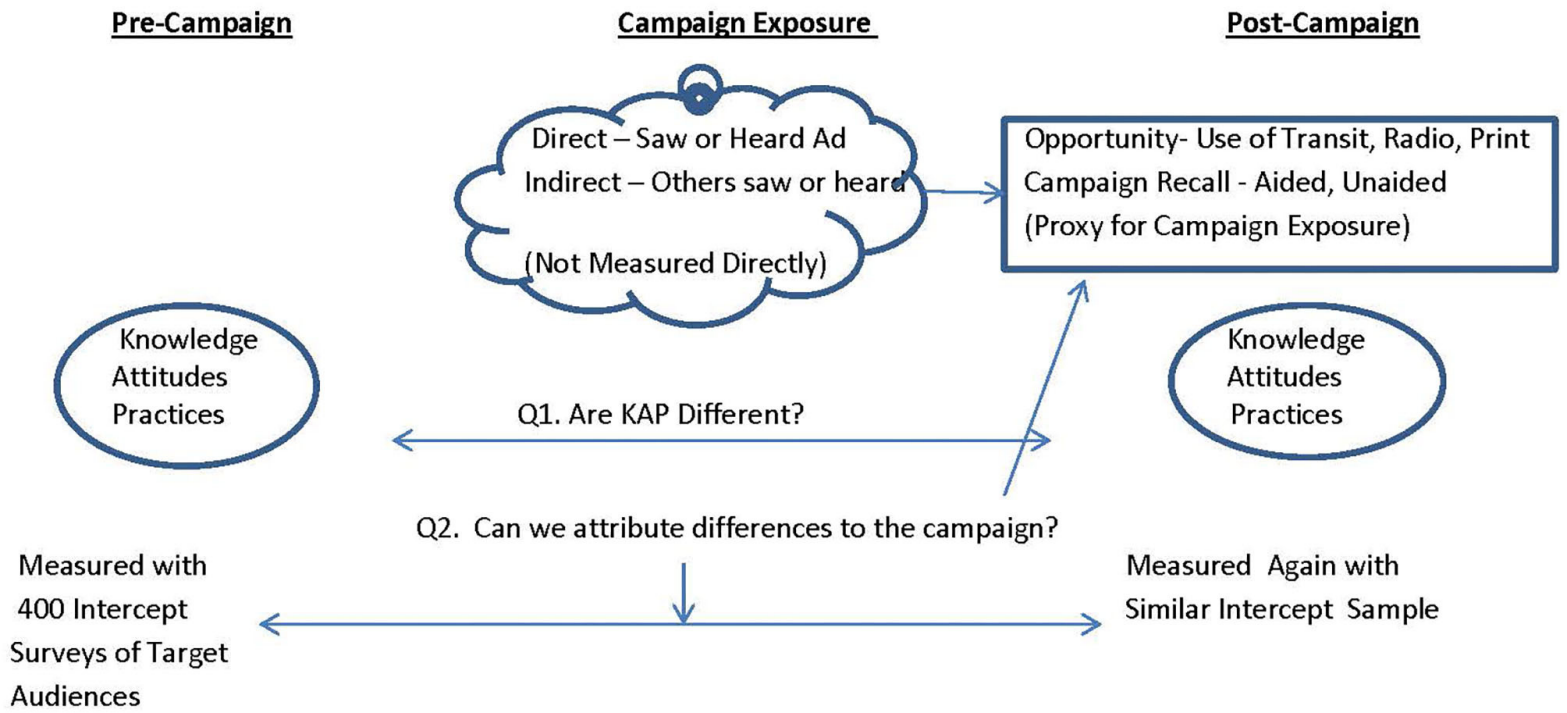
Evaluation design.

Figure 2 illustrates the evaluation design. The first goal of the evaluation was to determine if baseline and follow-up respondents differed on key knowledge, attitudes and practices related to salt and stroke. The second evaluation question was to determine, among follow-up respondents, whether those with greater campaign exposure (via use of transit, print media, or radio) were more likely to recall campaign elements, and have knowledge reflective of the campaign messages.

### Analysis

All paper surveys were visually edited, entered in to Qualtrics survey software, and analyzed with SPSS. Open-ended responses were grouped into structured response categories. The evaluation was reviewed and approved by the institutional review board of the Philadelphia Department of Health, with whom Drexel University has a reliance agreement.

Table 1 presents a comparison of respondent characteristics by wave (pre- or post-campaign), with the statistical significance of differences reported with Chi square and *T-*tests for categorical and continuous measures respectively. Table 2 presents descriptive analyses of respondent-reported opportunities for campaign exposure among post-campaign respondents, with comparisons by age, gender and education level, and statistically significant differences reported with Chi square statistics. Table 3 reports the association of predisposing, enabling and reinforcing characteristics (18) of post-campaign respondents to key evaluation metrics, including process (campaign familiarity), and outcome (key message knowledge) evaluation metrics.

**Table 1:** Comparison of Pre- and Post-Campaign Respondents by Demographics.

	**Baseline**		**Follow-up**		
	**Number of respondents**	**%**	**Number of respondents**	**%**	***P*-value for significance of difference^**a**^**
	(*n =* 400)		(*n =* 403)		
**Age**					
<35	12	3.0	13	3.2	0.66
35–44	150	37.5	137	34.0	
45–55	218	54.5	227	56.3	
56+	20	5.0	26	6.5	
**Gender**					
Male	189	47.3	195	48.4	0.75
Female	211	52.8	208	51.6	
**Level of formal education**					
No high school or G.E.D.	35	8.8	68	16.9	0.001
High school graduate or G.E.D.	173	43.5	175	43.4	
Any college or graduate school	190	47.7	160	39.7	
**Country of birth**					
United States	386	96.5	395	98.0	0.19
Another Country	14	3.5	8	2.0	
**Household composition**					
Lives alone	135	33.8	136	33.8	0.98
Lives with others	265	66.3	266	66.2	
**Current employment status**					
Working	234	58.5	200	49.6	0.01
Not working	166	41.5	203	50.4	
**Self-described race**					
Black or African American	365	91.3	370	91.8	0.34
Other Race (refusals, human race)	35	8.8	33	8.2	
**Hispanic ethnicity**					
Yes	30	7.5	15	3.7	0.02
No	369	92.3	387	96.3	
**Ever told had hypertension**					
Yes	195	48.8	198	49.1	0.91
No	205	51.3	205	50.9	
**Mean body size**^**b**^ **(mean and standard deviation)**					
Overall	5.80	1.67	5.33	1.63	<0.001
Male	5.24	1.68	4.91	1.73	0.05
Female	6.30	1.49	5.72	1.44	<0.001

a statistical significance based on Chi Square statistic for all variables, except for Mean Body Size, which is based on T-Test.

b interviewer rating of body size, from 1 (very thin) to 10 (very heavy), using the Stunkard et al. (17) Visual Rating Scale. At follow-up, 1 respondent did not answer household composition, and 2 respondents were not assessed for body size.

**Table 2:** Opportunities for Campaign Exposure and Access to Health-Related Media, Among Post-Campaign Intercept Survey Respondents, 2015 (*n* = 403).

	**All %**	**By gender %**			**By age %**			**By education %**		
		**Men**	**Women**	*P-*Value^*^	**<45**	**45+**	*P-*Value^*^	**≤HS**	**College**	*P-*Value^*^
Radio exposure (6 stations used in campaign)										
Listens several hours most days	66	69	63	0.30	66	66	0.64	67	65	0.66
Listens at least weekly	23	20	26		25	22		21	25	
No radio	11	11	11		9	12		12	10	
Transit exposure (rides weekly or more)										
Subway	70	76	63	<0.001	70	69	0.91	72	66	0.20
Bus	73	76	70	0.19	75	72	0.54	76	68	0.07
Print news exposure (4 weekly papers used for campaign)										
Ever Reads	83	84	82	0.53	81	84	0.53	82	83	0.71
General social media habits										
Uses internet	79	79	79	1.00	89	73	<0.001	72	91	<0.001
Goes to health websites	43	36	51	0.003	48	41	0.16	34	58	<0.001
Has smart phone	67	68	67	0.86	76	62	0.002	60	77	<0.001
Uses facebook	59	57	61	0.39	72	51	<0.001	53	69	0.002
Uses twitter	20	20	20	1.00	28	16	0.004	15	27	0.004
Uses instagram	27	23	32	0.04	42	19	<0.001	27	36	0.001
Food fit philly (FFP) specific										
Has heard of FFP	42	36	48	0.02	51	37	0.004	36	51	0.003
Knows it is a program of philadelphia city government	14	13	15	0.54	13	15	0.58	11	18	0.05
Knows it is a program of philadelphia department of public health	5	5	5	0.76	6	4	0.46	3	8	0.06

* P-Value based on the chi square test.

**Table 3:** Predisposing, Enabling, and Reinforcing Influences on Prompted Recall of “Mom's Says” Radio Campaign Content and Knowledge of Salt to Stroke Relationship, among Post-Campaign Intercept Survey Respondents, Philadelphia, 2015 (*n* = 403).

**Measure**	***N***	**%**	**Recalls salt and/or stroke content from “Mom Says” (*n =* 69, 17%)**		**Names stroke as problem from too much salt (*n =* 165, 41%)**	
			**Crude O.R.95% C.I**.	**Adjusted O.R.95% C.I**.	**Crude O.R.95% C.I**.	**Adjusted O.R.95% C.I**.
Predisposing characteristics						
Age 45 + vs. <45	253	63	2.22	2.65^***^	1.11	0.95
			1.22, 4.04	1.35, 5.17	0.74, 1.68	0.60, 1.52
Any college vs. ≤ HS	160	40	0.97	1.01	1.26	1.51^*^
			0.57, 1.67	0.55, 1.86	0.84, 1.90	0.96, 2.38
Male vs. female	195	48	0.79	0.68	1.19	1.51^*^
			0.47, 1.33	0.37, 1.25	0.80, 1.77	0.95, 2.38
Works now	200	50	1.13	1.19	0.96	0.87
			0.67, 1.90	0.66, 2.14	0.65, 1.43	0.56, 1.35
Lives alone vs. w/others	136	34	0.64	0.61	0.76	0.68^*^
			0.36, 1.15	0.31, 1.19	0.50, 1.17	0.42, 1.09
Uses internet	319	79	1.04	1.51	0.75	0.75
			0.55, 1.98	0.62, 3.69	0.44, 1.22	0.40, 1.42
Uses social media	248	61	0.97	1.03	0.89	1.06
			0.57, 1.64	0.51, 2.07	0.60, 1.35	0.63, 1.78
Enabling characteristics						
Listens to radio often	267	66	2.51	2.60^***^	0.90	0.74
			1.32, 4.78	1.31, 5.14	0.59, 1.37	0.47, 1.16
Rides transit often	327	81	0.66	1.01	0.63^*^	0.58^*^
			0.31, 1.21	0.50, 2.06	0.39, 1.05	0.33, 1.00
Reads newspapers	334	83	1.26	0.67	0.95	1.03
			0.75, 2.11	0.33, 1.39	0.56, 1.60	0.59, 1.79
Reinforcing characteristics						
goes to health websites	175	43	1.10	1.04	0.76	0.69
			0.66, 1.87	0.56, 1.93	0.51, 1.14	0.43, 1.10
Told by MD has HTN	197	49	1.65^*^	1.19	1.08	0.93
			0.98, 2.79	0.62, 2.27	0.73, 1.61	0.56, 1.52
Told by MD family member has HTN	268	66	1.24	1.15	1.03	1.02
			0.70, 2.20	0.63, 2.11	0.68, 1.57	0.66, 1.59
Told by MD to reduce salt	197	49	1.66^*^	1.71	1.05	0.98
			0.98, 2.81	0.88, 3.30	0.70, 1.56	0.59, 1.60
Body size of 6–9 vs. 1–5	203	50	1.26	1.03	1.27	1.31
			0.75, 2.11	0.56, 1.84	0.86, 1.90	0.85, 2.05
Mediating influence: recalls salt and/or stroke content in “mom says”	68	17			2.31^***^	2.34^***^
					1.36, 3.91	1.33, 4.10

* p < 0.10,

*** p < 0.01.

Of the six campaign elements described above (three print messages and three radio messages), the radio segment “Mom Says” was reported as familiar by the greatest number of respondents, and was therefore selected as the most useful measure of campaign recall for these analyses. Respondents were given a brief description of the segment's story line to aid recall (a man and his daughter were playing basketball, and the daughter was telling her father what to eat), and then through six open-ended questions, asked to further describe the story and its key messages. To quantify depth and accuracy of recall, beyond simple recognition of the ad, response content was coded for accurately recalling the story line as well as mentioning salt-related and/or stroke-related content. Analyses in Table 3 use a dichotomous measure of campaign familiarity, based on whether respondents mentioned either salt or stroke-related content. Campaign impact was measured by whether respondents named stroke as a possible health consequence of eating too much salt.

Predisposing characteristics are existing attributes such as age, gender and education level, or habits such as media use, which might determine whether an individual was likely to be influenced by the campaign. Enabling factors such as radio, newspaper, or transit use increase the likelihood of campaign exposure, and reinforcing factors may increase the campaign's salience or impact; for example, a heightened perception of susceptibility due to hypertension or obesity.

Odds ratios (O.R.) and 95% confidence limits (95% C.I.) for the relationship between respondent characteristics and the evaluation metrics are presented in Table 3. First bivariate, or “crude” odds ratios are presented, and then multivariate odds ratios are presented, with adjustment for all other covariates in the table.

## Evaluation Results

Both waves of intercept surveys were completed over 7 weeks. Although no formal denominator and response rate can be calculated for intercept surveys, 53% (1,106/2,098) of those approached completed screening, and 98% (804/817) of screened and eligible respondents completed the survey, yielding an overall response rate of 38% among all persons approached. Six percent of ineligible persons lived outside of Philadelphia, and 94% disclosed that they were outside the age range on initial screening.

Table 1 describes the characteristics of respondents, and compares them across the two waves. In both waves, data collectors successfully balanced the sample between men and women. Although over 90% of respondents were in the target age range (35–55), a small proportion stated during initial screening that they met age criteria, but disclosed that they were slightly younger or older when asked demographic information at the end of the survey. The majority of respondents had not attended college, and most were born in the United States. About 60% were currently working, with the remaining 40% not currently working, either because they were in school, unemployed, disabled or something else. The respondents also reflected other important characteristics of the general African American and US population, relevant to diet, health, and chronic disease risk. About half had been told by a physician that they had hypertension, and the average interviewer-assessed body size was slightly over the midpoint of five.

Compared to the baseline respondents, follow-up respondents were significantly likely to be thinner (especially women), significantly less likely to have college or graduate education, and less likely to be working or identify as Hispanic.

Table 2 describes the overall media-related behaviors of follow-up respondents, which may suggest their potential exposure to the campaign. Radio listening was relatively high, with two thirds of respondents reporting several hours of exposure on most days to at least one of the six stations airing the campaign messages. Approximately 70% of respondents use subway and 73% use public buses at least weekly, with women less likely to use the subway, and respondents with more education slightly less likely to use the bus. Readership of the four free weekly papers was high across all groups (83%). Therefore, as a whole, this group of respondents had frequent opportunity for exposure to the campaign.

In terms of general internet and social media habits, 79% of respondents used the internet, with significantly less use among older respondents, and those with less education. Women and college-educated respondents were more likely to report seeing information on health related websites. Access to smartphones was high (67%), with higher rates among younger and college-educated respondents, and use of Facebook showed similar patterns. Twitter and Instagram use was less common (20% Twitter, 27% Instagram), with use again higher among younger and more educated respondents. In addition, women were more likely than men to use Instagram.

When asked specifically about their familiarity with the nutrition-focused Food Fit Philly activities of the Philadelphia Department of Public Health (foodfitPhilly.org), slightly less than half (42%) of respondents claimed to have heard of it. Significantly more women (48 vs. 36%), respondents age 44 or younger (51 vs. 37%) and respondents with college education (51 vs. 37%) had heard of this initiative. However, a much smaller proportion (14%) could correctly identify it as a program of the Philadelphia city government, and only 5% knew it was a program of the Philadelphia Department of Public Health, with college-educated respondents marginally more likely to correctly attribute this program to its source. More commonly, respondents attributed the Food Fit Philly program to then-First Lady, Michelle Obama, who was a popular spokesperson for healthful diet and physical activity during the study period.

Table 3 presents process and outcome evaluation results. Overall, 17% of respondents spontaneously mentioned the salt- and/or stroke-related content in the “Mom Says” radio spot. (Thirty percent of respondents claimed to have heard of the spot, but of those, 13% did not mention salt or stroke as message content). Adjusted analyses for the first outcome show that, when controlling for all other variables in the model, older respondents were significantly more likely to recall salt and/or stroke-related content of this ad (O.R. 1.65, 95% C.I. 1.35, 5.17). In addition, respondents who reported listening to the radio more often were more likely to recall the spot's content (O.R. 2.60, C.I. 1.31, 5.14). No other predisposing, reinforcing, or enabling variables were associated with greater likelihood of recalling this key content.

The second set of analyses in Table 3 identifies several variables associated with post-campaign respondents' ability to mention stroke without prompting, as one problem that is associated with excess salt consumption. Overall, 41% of respondents mentioned “stroke.” This overall post-campaign frequency of mentioning stroke is substantially greater than at baseline, when only 17% of respondents mentioned stroke in answer to the same question (*p* < 0.001, data not shown).

Respondents with college education showed a trend toward greater ability to name stroke as a salt-related health issue (O.R. 1.51, 95% C.I. 0.96, 2.38, *p* < 0.10), and those who lived alone were less likely to do so (O.R. 0.68, 95% C.I. 0.42, 1.09), *p* < 0.10). However, results also demonstrate that respondents who recalled salt and/or stroke message in the “Mom Says” radio campaign were significantly more likely to independently list stroke as an outcome of excess salt consumption (OR 2.34, 95% CI 1.33, 4.10), suggesting that the campaign may have had a possible independent relationship to this knowledge.

## Discussion and Limitations

This analysis demonstrates that it is feasible to use relatively modest resources to evaluate public health communication campaigns which are targeted for populations at higher risk for chronic disease. In this campaign, low resource African American residents of Philadelphia were the intended audience for increasing knowledge of the relationship between excess sodium consumption and stroke. The socio-demographics of the central intercept-recruited participants in the two waves of data collection suggest that the evaluation design was successful in finding and successfully engaging members of the campaign's intended audiences.

The repeated cross-sectional evaluation design limits attribution of causality, as it could not determine if any given individual's knowledge or attitudes changed due to campaign exposure. However, it avoided campaign priming issues inherent in repeated measurement designs. Priming occurs when a baseline survey sensitizes respondents to the content of the campaign, and thus enhance the campaign's impact and bias follow-up responses. Moreover, it was a feasible evaluation strategy, compared to the resources required to successfully proportionally sample and recruit a respondent cohort, who would likely be challenging to relocate and re-interview post-campaign, without substantial loss to follow-up.

Furthermore, the results indicate that the media campaign itself was successful in reaching many of the sampled participants, as 30% reported familiarity with one of the most engaging campaign elements, a humorous radio story about a father and his daughter. Of those who remembered hearing the story, over half (17% of all respondents) could recall the main message without prompting. Given that ad recall was highly associated with radio listenership, results suggest that these respondents did have exposure to the campaign, and retained at least one element of its message, providing evidence of campaign-related effects (19).

However, because these interview data are not longitudinal, it is possible that the opposite effect occurred for some respondents. It is plausible that persons already aware of the connection between salt and stroke found the “Mom Says” spot more salient and were more likely to retain and recall its key health message. However, it could be argued that, as mediated health communication campaigns compete in a crowded media environment, successful campaign impact can be achieved by reinforcing key messages, as well as introducing them for the first time.

As described in the introduction, this campaign was just one element of a more comprehensive set of public health initiatives to reduce sodium intake in Philadelphia. In population-level public health initiatives, combining complementary elements at multiple socio-ecological levels (20) arguably increases the impact of each, beyond what it might achieve if launched as a single strategy. For example, media campaign messages on low sodium prepared food choices were potentially leveraged by changes in the food environment, such as “cues to action” at the point-of-purchase [i.e., Philadelphia's Healthy Chinese Carry Out campaign (15)]. Although the focus of this evaluation was the media campaign, a more comprehensive evaluation could identify both individual and synergistic impacts of the entire initiative. Additionally, historical effects, such as the contemporaneous national health initiatives mentioned by some respondents, may enhance or compete with specific campaign-related messages.

Finally, it is important to note that the campaign was active over a 10-month period, and our post-campaign evaluation measured recall ~6–13 weeks post-campaign. There were no significant differences in our key outcomes by week across that 7-week data collection window, but the study was not specifically designed to examine decay.

## Conclusion

These findings suggest that awareness of stroke and its causes remains relatively low among urban African-Americans, despite the prevalence of hypertension and overweight. Targeted media campaigns to raise awareness and introduce strategies for dietary behavior change are one important public health approach to reduce excess sodium in diets, and reduce population-level disparities in the burden of cardiovascular disease and stroke.

## Data Availability Statement

The raw data supporting the conclusions of this article will be made available by the authors, without undue reservation.

## Ethics Statement

The studies involving human participants were reviewed and approved by Institutional Review Board, Philadelphia Department of Public Health. The ethics committee waived the requirement of written informed consent for participation. Written informed consent was not obtained from the individual(s) for the publication of any potentially identifiable images or data included in this article.

## Author Contributions

AK led the design of the evaluation, development of measurement and analysis, and drafting of the manuscript. SS and AS contributed scientifically to all aspects of the work and reviewed the draft and final versions of the manuscript. UP, MS, JW, JL, AM, and JS participated in design and implementation of the data collection activities and contributed to the data analysis. AW secured funding for the project and oversaw all elements of the work. All authors reviewed the manuscript prior to submission.

## Conflict of Interest

The authors declare that the research was conducted in the absence of any commercial or financial relationships that could be construed as a potential conflict of interest.
